# Is Tobacco Use Associated with Neurocognitive Dysfunction in Individuals with HIV?

**DOI:** 10.1177/2325958218768018

**Published:** 2018-04-18

**Authors:** Billy Tsima, Sarah Jane Ratcliffe, Robert Schnoll, Ian Frank, Dennis Larry Kolson, Robert Gross

**Affiliations:** 1 Department of Family Medicine & Public Health, University of Botswana, Gaborone, Botswana; 2 Department of Biostatistics and Epidemiology, University of Pennsylvania, Philadelphia, PA, USA; 3 University of Pennsylvania Perelman School of Medicine, Philadelphia, PA, USA; 4 Center for Clinical Epidemiology and Biostatistics, University of Pennsylvania, Philadelphia, PA, USA

**Keywords:** HIV-1, HAND, smoking, tobacco, neurocognitive dysfunction

## Abstract

**Introduction:**

The prevalence of HIV-associated neurocognitive disorders continues to rise despite the widespread use of antiretroviral therapy. We aimed to define the risk of neurocognitive dysfunction among smokers relative to nonsmokers.

**Methods:**

We conducted a retrospective cohort study including HIV-infected adults ages 21 to 65 years. The Mental Alternation Test (MAT) was the primary outcome. The odds of cognitive impairment were compared using random-effects logistic regression to adjust for potential confounders.

**Results:**

Of 3033, 1486 (49%) were smokers. The odds ratio for the association between smoking and cognitive impairment was 1.12 (95% confidence interval: 0.85-1.49). Nonsmokers had a higher median MAT score relative to smokers (*P* = .01).

**Conclusion:**

There was no evidence that HIV-infected smokers had greater neurocognitive dysfunction relative to HIV-infected nonsmokers. While tobacco use remains an important health risk issue to address in the HIV population, it may not represent a risk factor for neurocognitive impairment.

## Introduction

The greatly improved life expectancy of people living with HIV/AIDS has been attributed to the widespread use of modern antiretroviral (ARV) regimens.^1^ The modern ARV medication era has resulted in a significant decrease in the prevalence of HIV-associated dementia, the most severe form of HIV-associated neurocognitive disorders (HAND).^1^ However, the prevalence of HAND, especially the milder forms, has increased.^1,2^ Although the severity of HAND has decreased with ARV use, the persistence of milder HAND forms may be due to central nervous system (CNS) toxicities of ARVs,^1,2^ persistent CNS inflammation, persistent injury to the blood–brain barrier during early stages of HIV infection, among other contributing factors.^3,4^ Thus, while the etiology of this increase remains unclear,^5–7^ evidence supports several risks.

HIV-infected individuals may be predisposed to cognitive impairment secondary to several factors.^8–13^ Heightened cardiovascular risk in HIV-infected individuals has been attributed to the effects of ARV medications on endothelial function^14,15^ as well as a rapid aging process.^16^ With HIV-infected individuals living longer in the modern ARV medication era, these mechanisms for cognitive impairment may become increasingly important.^17,18^

One potential cause of HAND that has been underexplored is cigarette smoking. The prevalence of cigarette smoking is estimated to be up to 3 times higher among people living with HIV/AIDS compared to the general US population.^19–25^ Chronic cigarette smoking is independently associated with a greater risk of HIV infection.^26^ However, it is known that cigarette smoking is associated with other addictive behaviors,^27^ and thus, smokers may be more likely to abuse illicit drugs and engage in unsafe sex, thereby placing them at greater risk of getting infected with HIV.

There is now a body of evidence indicating that chronic cigarette smoking is associated with increased risk of numerous biomedical conditions that may directly or indirectly compromise brain neurobiology and cognition in general population-based studies.^27^ Chronic smoking is associated with inferior performance on measures of general intelligence, visuospatial learning, and memory and fine motor dexterity.^28^ Subtle cognitive difficulties have been demonstrated in both young and older chronic smokers.^29^ Additionally, large population-based cohort studies have reported lower intelligent quotient scores among adolescent male smokers compared to nonsmokers.^30^ Despite this, there is a paucity of evidence from studies specific to HIV-infected individuals evaluating the relationship between smoking and cognition.

Enhanced nicotine metabolism in HIV-infected smokers is thought to result in increased reactive oxidative stress which, in turn, leads to an increase in viral replication.^31^ Furthermore, nicotine potentiates HIV expression in microglial cells, which may result in increased neuronal dysfunction.^32^ The transport of peripheral blood monocytes and activated CD4^+^ T lymphocytes carrying HIV across the blood–brain barrier into the CNS is a postulated mechanism for the development of HAND.^33–35^

The existing literature examining the association of smoking with cognition is limited to general population-based samples and conclusions remain inconsistent.^27^ The few studies in HIV-infected populations are limited to relatively small sample sizes and are mostly cross-sectional in design. These studies also report conflicting results in terms of the association between smoking and cognition. One US-based study is a notable exception for attempting to evaluate the effect of cigarette smoking on multiple domains of cognitive functioning in individuals with HIV.^36^ However, similar to other prior studies, the study lacked adequate power to test important differences. We therefore aimed to compare the risk of neurocognitive dysfunction between HIV-infected smokers and nonsmokers using a large clinical cohort of HIV-infected patients in care.

## Methods

### Study Design

We conducted a retrospective cohort study using routinely collected data from the University of Pennsylvania Center for AIDS Research (Penn CFAR). The Penn CFAR Adult/Adolescent Database and Specimen Repository was initiated in November 1999 to track demographic, clinical, and laboratory data from HIV-infected patients cared for at University of Pennsylvania affiliated hospitals. Participants in the CFAR Adult/Adolescent Database have laboratory-confirmed HIV, provide informed consent, and complete a standardized questionnaire that collects demographic, medical, and psychosocial data at enrollment.^37^ The database contains longitudinal data on demographics, clinical and therapeutics information, and behavioral and psychosocial data for over 3100 HIV-infected men, women, adolescents, and children. Ethical clearance to conduct the study was obtained from the institutional review board of University of Pennsylvania.

### Study Participants/Setting

HIV-infected adults between the ages of 21 and 65 years registered in the Penn CFAR database with data for 4 or more consecutive visits after enrollment were included. The minimum time between visits was 6 months. Therefore, 4 consecutive visits were required to ensure that participants were followed for at least 2 years. The time frame was selected because prior population-based studies have shown decline in cognition after follow-up periods of less than 2 years in smokers.^38^ We were interested in an adult population as the database captures adults in care.

### Variables of Interest

The primary exposure was self-reported use of cigarettes or other tobacco products over the past 1 month. We considered any use of cigarettes or tobacco products as exposure and defined smokers as those consistently reporting any use of cigarettes or tobacco products over the preceding 1-month period at each visit for at least 4 consecutive visits. Conversely, nonsmokers were defined as those consistently reporting nonuse of cigarettes or tobacco products over the preceding month at each visit for at least 4 consecutive visits. Participants who switched between smoking categories during follow-up were excluded from the analysis.

Our primary outcome was the Mental Alternation Test (MAT) score recorded during outpatient visits. The MAT was developed in the HIV clinical setting to screen for HAND.^39^ The test measures executive functioning, comprised of a cluster of higher order mental processes, which play a significant role in organizing, integrating, and maintaining other cognitive abilities.^39^ The MAT is correlated with other tests of cognition such as the Mini-Mental State Examination (MMSE)^40,41^ and is modeled on the Trail Making Test part B (TMT-B).^42^ The MAT consists of 3 tasks: (1) counting sequentially from 1 to 20, (2) reciting the alphabet, and (3) alternating between numbers and letters in sequential order for 30 seconds (eg, 1-A, 2-B, 3-C, etc). Scores reflect the number of correct alterations made during the third task and range from 0 to 51, with lower scores indicating lower executive ability. The test cutoff score of 15 yielded most accurate classification of abnormal results on both the MMSE and the TMT-B during validation studies.^39^

Other variables included education level, recorded as the highest grade of education completed (ie, less than high school graduate, high school graduate, some college, graduated from college, graduate or professional school after college), and HIV viral load (VL), dichotomized at 400 copies/mL. We used the 400 copies/mL cutoff for a definition of HIV VL suppression since our cohort enrollment started in 1999 using older technology than is currently available to detect lower HIV VL, and it is debatable whether lower levels of HIV VL are necessary for successful outcomes. Additionally, the following variables were included: age, history of alcohol and drug use, CD4 count, Center for Epidemiological Studies Depression (CESD) score,^43^ and sexual orientation.

### Data Analysis

Our primary end point was an MAT less than 15 points and our secondary end point was absolute MAT score. To account for longitudinal within-patient correlations between MAT scores assessed over time, random-effects logistic regression modeling was used. The analysis examined the relationship between the MAT score (dichotomized by a cutoff of 15 points) as a dependent variable and smoking status as an independent variable adjusted for age, gender, level of education, and CD4 nadir. We fitted a primary random-effects logistic regression model with the abovementioned variables (model 1) and a secondary model adding hypothesized variables with incomplete capture including HIV VL (model 2) and CESD score when available (model 3). We planned a priori to include HIV VL and CESD score, but extensive missing data precluded including them in the primary model. Additionally, we fitted model 1 using a different definition of smokers and nonsmokers, whereby the former were taken as those reporting everyday use of cigarettes or tobacco products while nonsmokers were defined as those reporting no use at all in the past month. All analyses were carried out using Stata 13.1 (Stata Corp).

## Results

A total of 3033 patients met the eligibility criteria for the study. Among these, 1486 (49%) were identified as smokers by our definition. The demographic, behavioral, and clinical characteristics of the patients at enrollment are presented in Table 1. Smokers were 3 years younger than nonsmokers, on average, based on age at enrollment. Both smokers and nonsmokers had similar rates of ever using alcohol. Smokers reported a history of ever using noninjectable drugs more often than nonsmokers. The median MAT score was 2 points higher in nonsmokers (median: 24, interquartile range [IQR]: 18-30) compared to smokers (median: 22, IQR: 16-30), and the difference was statistically significant (*P* = .01). When a cutoff point of 15 in the MAT was considered, the proportion of patients with MAT scores less than 15 at enrollment was significantly higher in smokers compared to nonsmokers. Among those patients with documented CESD scores, the CESD score at enrollment was similar in both groups. Smokers had lower CD4 count nadir compared to nonsmokers. The proportion of patients with suppressed HIV VL at baseline did not differ significantly among nonsmokers compared to smokers in patients with complete HIV VL information, as shown in Table 1.

**Table 1.** Baseline Demographic, Behavioral, and Clinical Characteristics by Exposure (Smoking).

Variable	Nonsmoker (n = 1547)	Smoker (n = 1486)	*P* Value
Age in years at enrollment, median (IQR)	52 (44-59)	49 (49-62)	<.001
Male sex, n (%)	1187 (77)	1135 (77)	>.5
MAT score at enrollment, median (IQR)	24 (18-30)	22 (16-30)	.03
CESD score at enrollment, median (IQR)	9 (4-19)	8.5 (3-19)	.4
CD4 count nadir, median (IQR)	337 (175-511)	267 (118-397)	<.001
HIV VL <400 copies/mL, n (%)	174 (12.6)	159 (13.2)	>.5
MAT score <15, n (%)	232 (16)	280 (20)	.03
Ever used alcohol, n (%)	1450 (94)	1486 (94)	>.5
Ever used injectable drugs, n (%)	274 (17)	230 (15)	.17
Ever used marijuana,^a^ n (%)	460 (30)	88 (6)	<.001
Ever used cocaine/methamphetamines,^a^ n (%)	291 (19)	43 (3)	<.001
Ever used heroin/opioids,^a^ n (%)	73 (5)	13 (1)	<.001
Used injectable drugs in the last month,^b^ n (%)	13 (1.0)	12 (0.8)	<.001
Ever used noninjectable drugs,^b^ n (%)	697 (45)	919 (62)	<.001
Used noninjectable drugs in the last month,^b^ n (%)	188 (12)	233 (16)	<.001
Sexual orientation			
Not documented, n (%)	21 (1.4)	8 (0.6)	>.5
Bisexual, n (%)	147 (9.7)	158 (10.6)	
Gay/lesbian, n (%)	616 (40)	619 (42)	
Heterosexual, n (%)	732 (47)	677 (46)	
Other, n (%)	31 (2)	24 (1.6)	

Abbreviations: CESD; Center for Epidemiological Studies Depression; IQR, interquartile range; MAT, Mental Alternation Test; VL, viral load.

^a^Missing = 76%.

^b^Missing = 25%.

The results of the random-effects logistic regression model adjusted for age at enrollment, gender, CD4 count nadir, and highest educational level at enrollment are shown in Table 2. Once we controlled for confounding factors, the odds of cognitive impairment were 12% higher among smokers, although the association was not significant. A similar association between smoking and MAT score was found after adjusting for HIV VL in the subset of patients who had complete VL information during follow-up, as shown in Table 3. The magnitude of the effect of smoking on MAT score was greatest for the model using the subset for which CESD score could be included (Table 4). A positive association between smoking and cognitive function was evident from the analysis that compared participants with the greatest smoking severity versus those with the least smoking exposure (Table 5).

**Table 2.** Model 1: Random-Effects Logistic Regression Model.

MAT Score Less Than 15	OR	95% Confidence Interval	*P* Value
Smoker	1.12	0.85-1.49	.41
Enrollment age	1.02	1.01-1.04	.002
Male gender	0.92	0.69-1.24	>.5
CD4 nadir	0.99	0.99-1.00	.11
Highest educational level at enrollment (base case: less than high school graduate)			
High school graduate or GED	0.46	0.34-0.64	<.001
Some college	0.25	0.17-0.37	<.001
Graduated from college	0.11	0.06-0.19	<.001
Graduate or professional school after collage	0.06	0.02-0.14	<.001

Abbreviations: GED, General Education Diploma; MAT, Mental Alternation Test; OR, odds ratio.

**Table 3.** Model 2: Random-Effects Logistic Regression Model for Subset of Patients with Viral Load Information.

MAT Less Than 15	OR	95% Confidence Interval	*P* Value
Smoker	1.07	0.68-1.68	>0.5
Enrollment age	1.02	0.99-1.06	0.12
Male gender	1.00	0.63-1.58	>0.5
CD4 nadir	0.99	0.99-1.00	>0.5
VL (log base 10)	0.99	0.90-1.08	>0.5
Highest educational level at baseline (base case: less than high school graduate)			
High school graduate or GED	0.40	0.13-1.26	0.12
Some college	0.17	0.02-1.19	0.08
Graduated from college	0.06	0-1.00	0.05
Graduate or professional school after collage	0.04	0-0.99	0.05

Abbreviations: GED, general education diploma; MAT, Mental Alternation Test; OR, odds ratio; VL, viral load.

**Table 4.** Model 3: Random-Effects Logistic Regression for Subset of Patients with Viral Load and CESD Information.

MAT Less Than 15	OR	95% Confidence Interval	*P* Value
Smoker	1.62	0.47-5.61	.44
Enrollment age	0.95	0.89-1.01	.11
Male gender	1.00	0.27-3.60	>.5
CD4 nadir (per cell)	1.00	0.99-1.00	.11
VL (log base 10)	0.96	0.74-1.24	>.5
Highest educational level at baseline (base case: less than high school graduate)			
High school graduate or GED	0.41	0.09-1.83	.24
Some college	0.08	0.010-0.58	.01
Graduated from college	0.11	0.009-1.29	.08
Graduate or professional school after collage	0.23	0.02-2.87	.25
CESD score	1.00	0.96-1.05	>.5

Abbreviations: CESD, Center for Epidemiological Studies Depression; GED, general education diploma; MAT, Mental Alternation Test; OR, odds ratio; VL, viral load.

**Table 5.** Random-Effects Logistic Regression Considering Smoker as Self-Reported Use of Cigarettes or Tobacco Products Every Day in the Past Month and Nonsmoker as No Use at All.

MAT Less Than 15	OR	95% Confidence Interval	*P* > z
Smoker	2.09	1.27-3.46	.004
Enrollment age	1.00	0.98-1.03	>.5
Male gender	1.01	0.58-1.77	>.5
CD4 nadir	1.00	0.99-1.00	>.5
Highest educational level at baseline (base case: less than high school graduate)			
High school graduate or GED	0.33	0.169-0.667	.002
Some college	0.13	0.05-0.29	<.001
Graduated from college	0.06	0.02-0.18	<.001
Graduate or professional school after collage	0.02	0.00-0.1	<.001

Abbreviations: GED, general education diploma; MAT, Mental Alternation Test; OR, odds ratio.

## Discussion

We found that HIV-infected smokers were no more likely to have clinically significant cognitive impairment than HIV-infected nonsmokers. After controlling for confounding factors, the elevated risk was only 12% higher and not statistically significant. Other researchers have reported a negative association between smoking and cognition in HIV-infected patients in care.^36^ Bryant et al found that smokers had poorer cognitive function than HIV-infected past smokers in the domains of auditory verbal learning and memory, visuospatial memory, overall cognitive efficiency, executive skills, processing speed, and working memory.^36^ They used a battery of neuropsychological tests to measure cognition in contrast to our study, which was limited to the use of MAT as a measure of cognition. Although these researchers similarly used self-reports to categorize smokers, they used arbitrary groups of current smokers, past smokers, and never smokers without clear description of these categories. As these were self-reported, it is possible that exposure misclassification biased their findings.

In our sensitivity analyses, wherein we included the subset of patients with complete VL information, a similar, although of lesser magnitude, association between smoking and MAT score was revealed. The results of the sensitivity analyses confirm the primary finding of the lack of a strong association between smoking and neurocognitive dysfunction in HIV-infected smokers.

We did not consider the level of smoking in terms of the number of cigarettes consumed per day among the participants, as this information was unavailable. However, we estimated the level of smoking by the self-reported frequency of smoking (eg, “every day,” “not at all”). Since the level of exposure may have been variable among the smokers in our study, with possibly more mild to moderate smokers than heavy smokers, our finding may reflect the effect of mild to moderate smoking rather than heavy smoking. It is therefore possible that the difference in the proportion of participants with cognitive impairment is greater with greater smoking exposure. There is evidence from in vitro studies that suggests that smoking leads to an increase in HIV replication.^44^ Therefore, the mechanism by which smoking could lead to cognitive impairment in HIV-infected individuals could be through this pathway wherein tobacco use enhances HIV replication in a dose-dependent manner, which in turn affects cognition. Furthermore, our analyses did not consider the duration of smoking as the information was not available. Nicotine from tobacco use is known to enhance alertness and cognition in acute use, although its chronic use is associated with cognitive decline.^27,28^ It is therefore possible that in our study, the proportion of participants who had started smoking proximal to the data collection (new smokers) was significantly higher compared to chronic smokers. Our finding of a lack of association between smoking and cognitive impairment measured by the MAT could be influenced by a high representation of new smokers, who are likely to perform better than both nonsmokers and chronic smokers in our cohort due to the recognized cognitive enhancement of acute versus chronic cigarette smoking.^27,28^ Our definition of smoker and nonsmoker was based on self-reported use of cigarettes or tobacco products in the past month. This may not have adequately differentiated smokers from nonsmokers. In the sensitivity analyses, we considered smokers as only those who report the greatest smoking severity (reported “every day” use of cigarettes or tobacco products in the past month) and nonsmokers as those with the least exposure (reported “not at all” use of cigarettes or tobacco products in the past month). In contrast to our primary analyses, this suggested an association between smoking and cognitive function. Thus, it appears that the definition of smokers and nonsmokers may have affected our findings. This suggests that the conflicting reports in the literature from studies investigating the association between smoking and cognitive function in HIV-infected individuals may be due to how smokers and nonsmokers are defined in these studies or whether the amount smoked is considered.

Despite the lack of an association between smoking and cognitive dysfunction in our study, close to half of the participants in this cohort study were identified as smokers. This high prevalence of smoking in HIV-infected patients is consistent with other studies from the United States and raises concern regarding its potential morbidity and mortality burden. It is therefore crucial that efforts to control the HIV epidemic address the issue of smoking in this population by seeking novel ways that provide therapeutic interventions for HIV-infected smokers. The development of biomarkers that could help diagnose and predict the presence and severity of HIV-related brain diseases such as HAND has been recognized as a critically important and largely unmet clinical need.^42^ Such biomarkers will possibly help in better quantifying the neurological effects of heavy smoking among HIV-infected individuals.

Our study has several strengths and limitations. The MAT has utility in clinical settings and has the advantage that it does not require specialized training to administer unlike many other neuropsychological tests developed for measuring cognitive impairment. However, the MAT should be interpreted cautiously since a range of factors including depression could affect performance.^45^ The retrospective design of our study resulted in missing data due to incomplete records. This limitation, inherent to retrospective studies, may be a source of misclassification bias since the extent of missing data may differ between the 2 groups. The missing data for VL and CESD score led to the exclusion of these variables in the primary model. However, sensitivity analyses including these variables did not change the results significantly and thus confirmed the results of the primary model. The MAT is limited to testing executive functioning and does not test all the domains that comprise cognitive function. It is used as a screening test for HAND and does not diagnose cognitive dysfunction. Comprehensive tests for cognition include batteries of neuropsychological assessments. Although these tests are the “gold standard,” the long periods of assessment required to administer such batteries of tests possibly impose restriction on conducting studies investigating cognitive impairment using clinical cohorts. Additionally, the number of participants enrolled in such studies is limited owing to the long testing time required. Shorter screening tests that assess cognitive domains often affected by HIV such as executive function and attention/concentration are useful alternatives to these batteries. Although the MAT is not comprehensive in this regard, it screens for important domains, which are critical in the care of HIV-infected individuals. As a measure of executive functioning, comprised of a cluster of higher order mental processes, which play a significant role in organizing, integrating. and maintaining other cognitive abilities, the MAT is a reasonable estimate of cognition function when neuropsychological batteries are not feasible.^46^ Our results may be confounded by known and unknown unmeasured factors. Our exposure of interest was measured through self-reports and may not be accurate and may have resulted in exposure misclassification bias as smoking status may be underreported. Furthermore, the frequency of smoking as assessed by the existing questions in the database is likely to be inaccurate and remain subjective given the nature of self-reports. However, self-reports of smoking status have been shown to be accurate in many studies.^21,47^

## Conclusion

There was little evidence that HIV-infected smokers had greater neurocognitive dysfunction relative to HIV-infected nonsmokers. Thus, while the prevalence of smoking indicates that it remains a critical clinical issue for HIV-infected patients, additional causes of neurocognitive impairment in this population should be explored.
